# Estimating the basic reproduction number for the 2015 bubonic plague outbreak in Nyimba district of Eastern Zambia

**DOI:** 10.1371/journal.pntd.0008811

**Published:** 2020-11-09

**Authors:** Joseph Sichone, Martin C. Simuunza, Bernard M. Hang’ombe, Mervis Kikonko

**Affiliations:** 1 Department of Disease Control, School of Veterinary Medicine, University of Zambia, Lusaka, Zambia; 2 School of Health Sciences, University of Zambia, Lusaka, Zambia; 3 Africa Centre of Excellence for Infectious Diseases of Humans and Animals, University of Zambia, Lusaka, Zambia; 4 Department of Paraclinical Studies, School of Veterinary Medicine, University of Zambia, Lusaka, Zambia; 5 Department of Mathematics and Statistics, School of Natural Sciences, University of Zambia, Lusaka, Zambia; University of Zimbabwe Lake Kariba Research Station, ZIMBABWE

## Abstract

**Background:**

Plague is a re-emerging flea-borne infectious disease of global importance and in recent years, Zambia has periodically experienced increased incidence of outbreaks of this disease. However, there are currently no studies in the country that provide a quantitative assessment of the ability of the disease to spread during these outbreaks. This limits our understanding of the epidemiology of the disease especially for planning and implementing quantifiable and cost-effective control measures. To fill this gap, the basic reproduction number, R_0_, for bubonic plague was estimated in this study, using data from the 2015 Nyimba district outbreak, in the Eastern province of Zambia. R_0_ is the average number of secondary infections arising from a single infectious individual during their infectious period in an entirely susceptible population.

**Methodology/Principal findings:**

Secondary epidemic data for the most recent 2015 Nyimba district bubonic plague outbreak in Zambia was analyzed. R_0_ was estimated as a function of the average epidemic doubling time based on the initial exponential growth rate of the outbreak and the average infectious period for bubonic plague. R_0_ was estimated to range between 1.5599 [95% CI: 1.382–1.7378] and 1.9332 [95% CI: 1.6366–2.2297], with average of 1.7465 [95% CI: 1.5093–1.9838]. Further, an SIR deterministic mathematical model was derived for this infection and this estimated R_0_ to be between 1.4 to 1.5, which was within the range estimated above.

**Conclusions/Significance:**

This estimated R_0_ for bubonic plague is an indication that each bubonic plague case can typically give rise to almost two new cases during these outbreaks. This R_0_ estimate can now be used to quantitatively analyze and plan measurable interventions against future plague outbreaks in Zambia.

## Author summary

Bubonic plague is a historically important and neglected bacterial infectious disease that has claimed more than 170 million lives from multiple devastating epidemics especially during mediaeval and earlier times. Despite a recorded decline in the disease for much of the 21^st^ century, incidence has begun to sharply rise again mainly in developing nations including Zambia which recently recorded a five-fold increase in recorded cases by the turn of the century. For developing and resource strained nations like Zambia, such major public health challenges need to be addressed with the most cost-effective, efficient, and sustainable approaches appropriate to the region. This firstly requires developing a sufficient and comprehensive epidemiological understanding of the disease. This study therefore estimated the basic reproduction number (R_0_) for bubonic plague which is an estimate of the average number of secondary infections expected to arise from a single infectious individual during their infectious period assuming the entire population is susceptible to the infection. Based on data from the Nyimba district bubonic plague outbreak in Zambia, we estimated R_0_ for the infection to range between 1.5599 to 1.9332 which shows that each infectious individual with bubonic plague can potentially infect up to two other individuals during their infectious period. We also observed that this R_0_ estimate was comparable to that previously estimated for large medieval bubonic plague epidemics. Estimating this important epidemiological parameter for bubonic plague greatly enhances existing knowledge of the epidemiology and transmission dynamics of the infection in Zambia and creates prospects for better management of the disease through informed planning.

## Introduction

Plague is a re-emerging infectious disease that has claimed more than 170 million lives in three waves of devastating pandemics in human history. The current third pandemic which begun in the late 1800s has spread the farthest globally and continues to affect vulnerable communities mainly in developing nations [1–4]. Plague results from infection by the gram negative bacterium *Yersinia pestis*. It primarily infects rodents and their fleas but infection can spread to humans causing outbreaks with bubonic plague being the most common clinical form [5–8]. In Zambia, plague was first recorded in 1917 in Tembwe village of Chama district in the now Muchinga Province. By 1956, about 247 cases and 205 deaths were recorded from over 13 separate outbreaks in the country [1,7]. This was followed by a general period of quiescence with fewer new outbreaks. By the turn of the century however, incidence began to sharply rise again in the country than previously seen. For example, between 1997 and 2007 alone, about 1,447 new cases were recorded from 4 major outbreaks which is more than five times that recorded from all previous outbreaks in the country put together [1,11]. Recent studies in Zambia have highlighted important ecological and socio-cultural factors, and life-style practices that precipitate the occurrence and rapid spread of these plague outbreaks [1, 9–11]. However, the outcome of these studies did not generally include a quantitative assessment of this rapid spread of the infection in relation to identified factors. This partly limits our understanding of the transmission dynamics of the disease especially for the purpose of developing quantifiable and cost-effective control measures. To help fill this gap, the basic reproduction number, R_0_, for bubonic plague was estimated in this study based on data from the latest 2015 Nyimba district bubonic plague outbreak in Zambia, for which data was available. R_0_ is defined as the average number of secondary infections arising from a single infectious individual during their infectious period in an entirely susceptible population [12–14]. It is the single most important epidemiological parameter that gives a quantitative measure of the transmissibility of an infectious disease in the population and is used to estimate the expected magnitude and extent of spread for an infectious disease outbreak [13,14]. More importantly, R_0_ is used to guide the magnitude of control measures that will be required to control the disease [14–18]. The basic reproduction number has been used to study various important infectious diseases including African swine fever-ASF [19,20], cholera [18,21], Severe acute respiratory syndrome-SARS [16], and foot and mouth disease-FMD [15], among others. The main aim of the current study was to estimate the basic reproduction number for bubonic plague in Zambia and briefly characterize its importance for understanding the transmission dynamics of plague outbreaks in the country. Estimating such important epidemiological parameters as the basic reproduction number for bubonic plague would increase the epidemiological understanding of the disease in the country and meaningfully contribute to the planning of control measures.

## Materials and methods

### Study area

Nyimba district is located in the Eastern province of Zambia [1]. In 2015, it experienced its first outbreak of bubonic plague in a small village but the village characteristics information was generally limited [11]. However, similar to other districts in the Eastern province; agriculture, hunting, and forestry comprise some of the main economic activities by which the communities sustain their livelihoods in the district [22]. Such activities increase interactions between the humans and wildlife which can increase chances of spread of zoonotic infections to humans [1,23]. Additionally, by 2010 Nyimba reached a population size of slightly over 85, 000 people giving about a 1.9 percent annual population growth rate from its population of about 70,425 people in the year 2000 [22]. The population density stood at about 8.1 persons per square kilometer with more than 80% of its population living in rural areas [22]. The average household size in the rural areas was about 5.3 members making it the second highest for the province [22]. This increase in the population size over the years and the consequent higher population densities in such plague endemic regions poses a threat for rapid spread of the disease due to overcrowding and increased human contact [1,9,10]. All these factors may have played a role in causing and propagating the bubonic plague outbreak in Nyimba analyzed in this study.

### Source of data

Online secondary epidemic incidence data for the 2015 Nyimba district bubonic plague outbreak in Zambia, made publicly available in 2016 [11], was retrieved and analyzed. This outbreak was selected as it had the most complete bubonic plague epidemic incidence data available for reliable analysis at the time of the study.

### Case definition and outbreak data

The Nyimba bubonic plague outbreak occurred between March 26^th^ and May 5^th^ 2015 [11]. During the entire outbreak, a total of 111 suspected cases were identified and evaluated for possible plague and all received antibiotic treatment [11]. Out of this total 111 suspected patients, 21 actual cases of plague were identified using case definition based on evidence of clinical illness compatible with bubonic plague as detailed in [11] and these were used for the analysis in our study. The median age for the cases was 8 years (range = 3–18 years) and all came from the same village. Ninety-five percent (20) were aged below 15 years old and 11 (52%) were male [11]. *Y*. *pestis* was detected in six (29%) of the cases through polymerase chain reaction (PCR). It was reported however that 12 of these cases actually tested positive for malaria during initial evaluation and this includes all six who tested PCR-positive for plague [11]. In fact, all of the first 4 cases (based on date of symptom onset) tested positive for malaria and were hence initially only treated with anti-malarial drugs on their first visit to the health center [11]. Three of these first 4 patients had the antibiotics included to their treatment regimen only on their second visits to the clinic. Two of the three patients who experienced this delayed initiation of antibiotic therapy died [11]. Apart from the antibiotic treatment of patients during the outbreak, indoor spraying of about 1,303 (96%) households in the affected catchment areas was conducted aimed at reducing the flea population, and recommendations were made to local leaders regarding risks for plague transmission [11]. The epidemic curve for this outbreak is given in the Morbidity and Mortality Weekly Report as published by Sinyange and colleagues [11] which shows that cases for the outbreak peaked by 6^th^–7^th^ April 2015 after which the incidence seemed to suddenly drop and the outbreak generally died out with the last case being recorded on 1^st^ May 2015.

### Analytical approach to the estimation of the basic reproduction number

Due to limited epidemiological data for the outbreak in Nyimba, the basic reproduction number, R_0_, for bubonic plague was estimated using a simplified mathematical method as described by others [12–14,19,20,39], which estimates R_0_ directly from the epidemic incidence data. This method was chosen as the primary method of estimation for the study as it is quick and robust even in the absence of abundant epidemiological data. To give credence to the results of this primary method, a second alternative method was also used to estimate R_0_ for the same bubonic plague outbreak in Nyimba by means of creating a complete compartmental mathematical model for the infection. Both these methods used however relied on the following two underlying assumptions made about the Nyimba bubonic plague outbreak: (1) Since various disease interventions were applied during the course of this outbreak (i.e antibiotic treatment of patients, indoor spraying, and sensitization), it was assumed in this study that the disease transmission must have occurred naturally in the community only during the early stages of the outbreak which caused the initial peak in cases around the 7^th^ of April 2015 [11,24–28]. For this reason, both methods of R_0_ estimation used in this study relied only on analysis of the data from the initial growth phase of the Nyimba bubonic plague outbreak leading up to its peak; i.e before the provided interventions took full effect [20,29]. (2) Further, it was also assumed in this study that during the outbreak, bubonic plague infection was transmitted from human to human through various human ectoparasites vectors (such as body lice and fleas) which are capable of transmitting plague [6,8,30–35,47]. In this model of transmission, infectious humans will first transmit bubonic plague infection to the human ectoparasites vectors and these infectious vectors will subsequently transmit the infection to other susceptible humans [6,36–38]. Note that current epidemiological knowledge indicates that modern bubonic plague outbreaks generally follow epizootics of rats and only later include ectoparasites [6]. However, as a report of an overt rat epizootic was generally not available for our study outbreak in Nyimba [11], it was assumed in this study that the study outbreak in Nyimba mainly followed this possible human to human transmission of the infection through human ectoparasites after the infection had been introduced into the human population.

### Method 1 (primary method): Estimation of R_0_ for bubonic plague using the epidemic doubling time and the average infectious period

Briefly, this method assumes that in a homogeneously mixing population of susceptible subjects, the number of new cases increases exponentially during the initial stages of the epidemic. The basic reproduction number is then estimated from the initial exponential growth rate of the epidemic based on the analysis of the initial segment of the epidemic curve [12–14,19,20,39]. According to [39], R_0_ in this natural system can be estimated using Eq 1 below:

$$Ro=1+(\frac{D}{Td})ln2$$ (1)

Where *Td* is the epidemic doubling-time, *D* is the average infectious period for bubonic plague, and *ln*2 is simply the natural log of 2. The epidemic doubling time (*Td*) was estimated using the exponential epidemic growth rate of the initial growth segment of the epidemic curve as summarized in the following steps: **Step 1.** A plot of the cumulative number of cases plotted against time for the period of initial rise of cases for the Nyimba bubonic plague outbreak was generated. An initial 17-day period from the 23^rd^ March–8^th^ April, 2015 was taken to approximately represent this period. **Step 2.** To assess the assumption of initial exponential growth of the outbreak, an exponential curve was fitted to the plot generated in step one. The R^2^ (coefficient of determination) value was used to assess how well the exponential model fit this observed data [20]. The fitted exponential model described the initial exponential growth rate of the epidemic through the following general expression for the exponential function:

$$C(t)\approx C(0)e^{\alpha (t)}$$

Where *C*(*t*) is the total number of cases at a given time (*t*), *C*(0) is the initial number of cases at the start time, *e* is the mathematical constant and *α* is the epidemic growth rate [40]. To obtain the 95% confidence interval for this estimated epidemic growth rate (*α*), a log transformation of the plotted epidemic curve in step one was made by simply plotting the natural log of the cumulative number of cases against time. This produced a linear relationship between the log cumulative number of cases and time. This linear relation was arithmetically derived by simply taking the natural log of both sides of the general expression for the exponential function given above to produce the following linear expression:

ln(C(t))=ln(C(0))+α(t)

Therefore, a linear regression model was run for this plot and the 95% confidence interval for the already estimated epidemic growth rate *α* (gradient) was obtained from the linear regression output. Here the R^2^ value was equally used to assess how well the linear regression model fitted the observed data [20]. **Step 3.** Using the epidemic growth rate and its 95% confidence interval estimated in step two above, the average epidemic doubling time was estimated using the relation between the epidemic doubling time and the epidemic growth rate given by the expression below [41]:

$$Td=\frac{ln2}{\alpha }$$

The 95% confidence interval lower and upper limits for the epidemic doubling time (*Td*) were therefore determined by the interval for the estimated epidemic growth rate (*α*).

For this primary method, the duration of infectiousness for bubonic plague infection was the only parameter that was obtained from literature. Despite estimations greatly varying in the literature, this parameter was simply estimated to range between three and five days. This simply represents the most central estimate of the average period that the infectious individual may pass the infection to susceptible human ectoparasites vectors which transmit the infection to other susceptible individuals before these vectors die from the infection themselves; assuming an early phase transmission cycle in the vectors [6–8,30,33, 35,42,43]. The estimated value of the epidemic doubling time and this approximate duration of infectiousness for bubonic plague were then used in Eq 1 to estimate R_0_ for bubonic plague. The 95% confidence interval lower and upper limits for R_0_ were therefore equally delimitated by the interval for the epidemic doubling time (*Td*). All calculations were done in Microsoft Excel Software.

### Method 2 (secondary method): Estimation of R_0_ for bubonic plague using the derived SIR deterministic compartmental mathematical model for bubonic plague

For the second method, a deterministic compartmental mathematical model was created for the Nyimba bubonic plague outbreak and this model was used to estimate the basic reproduction number. As the infection was assumed to be spread through vectors, the Ross Macdonald Susceptible-Infectious-Removed-Susceptible-Infectious model, denoted as SIR-SI, was initially created as a first step in the modelling process [38]. This model type, originally developed for malaria, is a standard mathematical model for vector-borne pathogens that tracks the infections in both the human and the vector populations [6,25–27, 36,37,38,44–46]. The parameter values used for creating the model were obtained from various literature and where such information could not be readily found the parameter values were estimated based on reasonable assumption and biological plausibility. Our model was then finalized by converting it into an amalgamated Susceptible-Infectious-Removed (SIR) version of the model which tracked the growth of the infection only in the human population; with the concomitant spread of the infection in the human ectoparasites vector population already accounted for in the model (assuming that infection dynamics in the vector population were fast compared to those of the human population) [44]. This version of the model was preferred because the main population of interest for this study was the human population and also because SIR models are simpler than SIR-SI models which makes analysis and parameter estimation easier [44]. Additionally, it may not always be necessary to explicitly incorporate the vector population when modeling the transmission of some vector borne diseases in some populations [44]. For example, in the study done by Pandey and others [44] a similarly derived SIR model was substantially better than the SIR-SI version of the model in explaining dengue hemorrhagic fever data from Thailand. Furthermore, for accurate estimation of R_0_ from the model, the model in this study was developed without the inclusion of disease interventions in order to simulate the transmission dynamics of bubonic plague infection for the period of initial growth of the Nyimba bubonic plague outbreak when the infection was thought to spread naturally in the community. For the purpose of this model, the following main assumptions were made about the disease transmission dynamics for the study outbreak:

1. The human population was initially completely susceptible to the infection.
2. Individuals in the population were considered to be homogenously mixing.
3. It was assumed that during the outbreak the infection was transmitted from human to human through various human ectoparasites vectors [6,8,30–35,47].
4. The human population was constant owing to the short duration of the Nyimba bubonic plague outbreak.
5. The infection is immunizing [6,23].
6. The human ectoparasites vectors do not recover from the infection.

**Fig 1:**
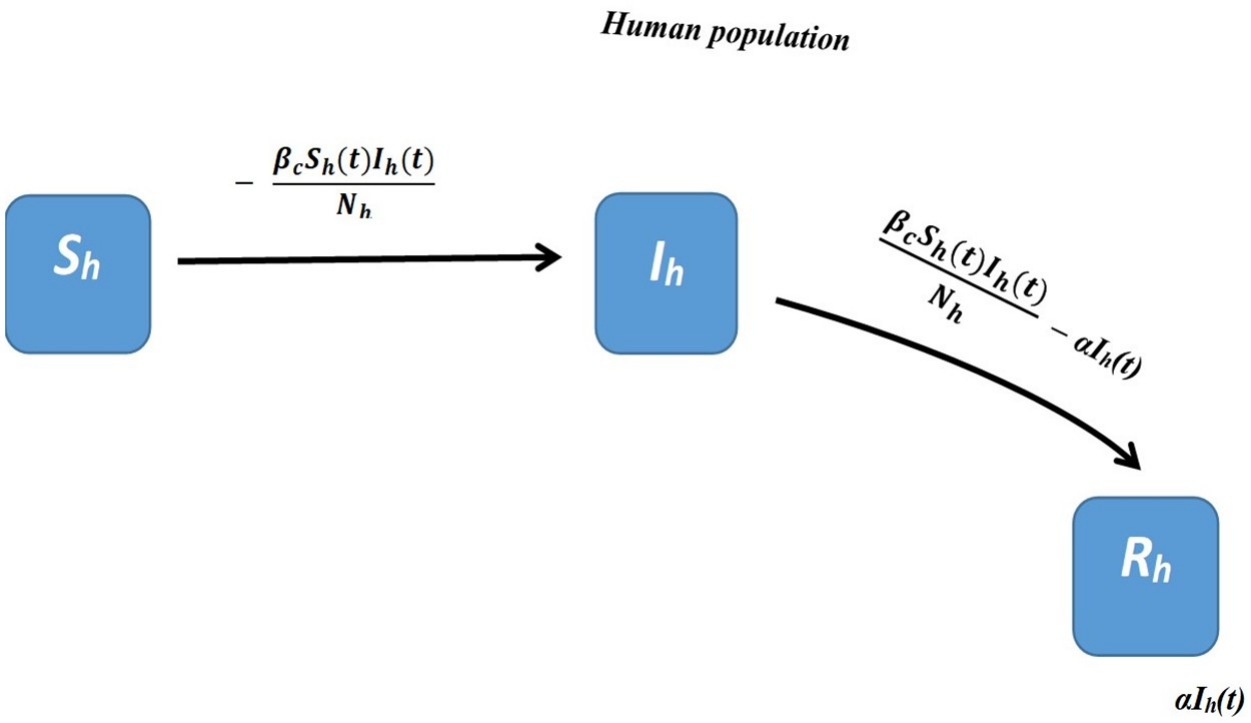
Schematic diagram for the derived SIR deterministic compartmental mathematical model system for bubonic plague disease.

Table 1 shows the parameters and variables used for the derived SIR model and a schematic representation of this disease model system is shown in Fig 1.

**Table 1:** Parameters and variables used for the derived SIR deterministic compartmental mathematical model system for bubonic plague disease outbreak.

Parameters and variables	Description	Value
*β*_*f*_	Transmission rate of bubonic plague from infectious human ectoparasites to humans per unit time. [6]	Range (0.05–0.123) was considered
*β*_*h*_	Transmission rate of bubonic plague from infectious humans to human ectoparasites per unit time. [6]	0.5
*α*^−1^	Duration for which infectious humans transmit the infection to susceptible human ectoparasites (days). *α* is recovery rate. [6–8,42]	4.5
*μ*^−1^	Duration for which infectious human ectoparasites transmit the infection to susceptible humans (days). [6,30,33,35]	5.5
*S*_*h*_ *(t)*	Total number of susceptible humans at a given time (t)	Is a function of time
*I*_*h*_ *(t)*	Total number of infectious humans at a given time (t)	Is a function of time
*R*_*h*_	Total number of removed humans at a given time (t) (this includes both those that die from the infection as well as those that recover)	Is a function of time
*N*_*h*_	The total human population (based on estimates by local health experts for the village of the outbreak)	350
*β*_*c*_	Composite human to-human transmission rate of the bubonic plague infection through various human ectoparasites vectors per unit time.	Given as $\beta_{c}=\frac{\beta_{f}\beta_{h}}{\mu }$

Note: Single values given as approximate point estimates.

### Eq 2 (for the derived SIR model for bubonic plague infection tracking the infection in the human population)

$$\begin{array}{lll} \frac{dS_{h}(t)}{dt}= & -\frac{\beta_{c}S_{h}(t)I_{h}(t)}{N_{h}} & \left(2\text{a}\right)\\ \frac{dI_{h}(t)}{dt}= & \frac{\beta_{c}S_{h}(t)I_{h}(t)}{N_{h}}-{\alpha \text{I}}_{\text{h}}(\text{t}) & \left(2\text{b}\right)\\ \frac{dR_{h}(t)}{dt}= & {\alpha \text{I}}_{\text{h}}(\text{t}) & \left(2\text{c}\right) \end{array}$$

### Derived SIR model explained

In the derived SIR model, the transmission of bubonic plague by the infectious humans through various human ectoparasites vectors is modeled by three equations as given in Eq 2 above. This model tracks the infection in the human population through the three compartments that are a function of time: susceptible (*S*_*h*_), infectious (*I*_*h*_), and removed (*R*_*h*_) humans. The constant total human population *N*_*h*_ is given as *N*_*h*_ = (*S*_*h*_) + (*I*_*h*_) + (*R*_*h*_) and this was estimated to be about 350 people for the village of the outbreak based on estimates by local health experts in Nyimba. Susceptible people in the population (*S*_*h*_) acquire the infection from the infectious people (*I*_*h*_) at the rate *β*_*c*_ for a period *α*^−1^. This transmission rate *β*_*c*_ is called the composite human to human transmission rate and it already accounts for the infection transmission dynamics in the intermediate human ectoparasites vectors [44]. The infectious humans leave the infectious compartment at the rate *α* to enter the removed compartment. It is assumed that all infectious individuals eventually either die from the infection at the end of this period *α*^−1^ or they may naturally recover from the infection but are all considered no longer able to transmit the infection [6,42]. The exact proportions of those that die from the infection and those that survive it in the removed compartment therefore is not described in the model but may depend on the typical case fatality rates for bubonic plague infection [8,7].

### Derived SIR model simulation

**Fig 2:**
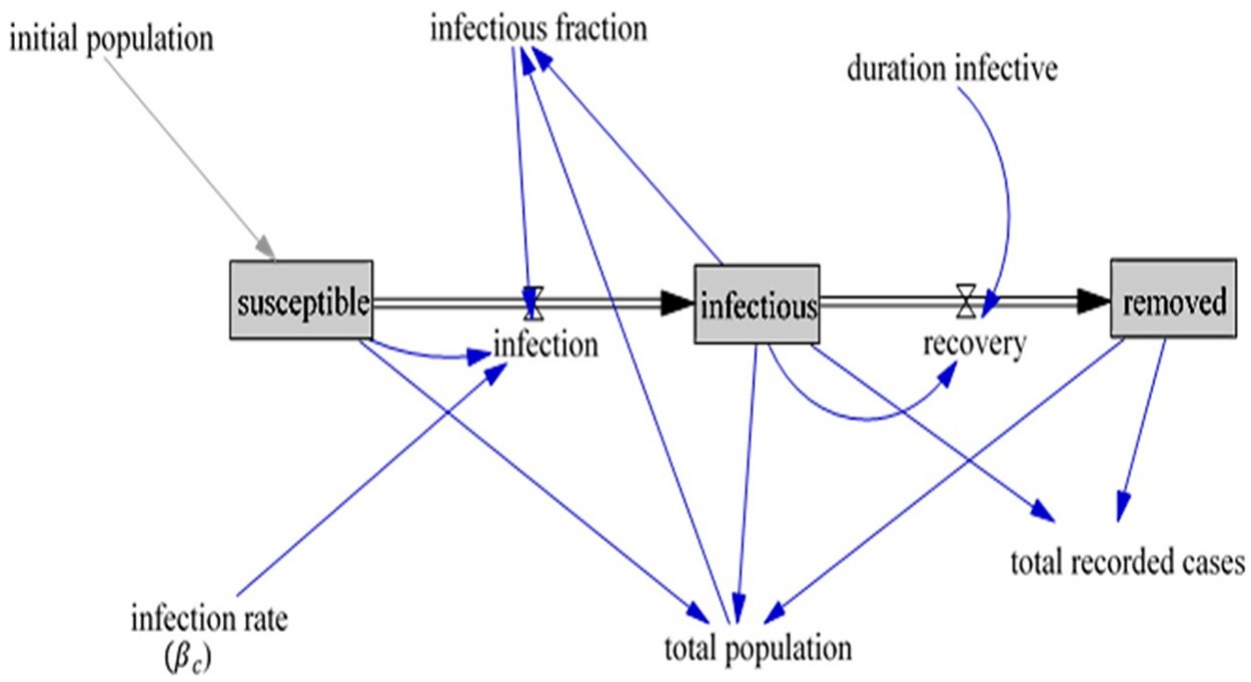
Flow diagram of the human population system for the derived SIR deterministic compartmental mathematical model for bubonic plague as generated in Vensim systems dynamics modeling software—Personal learning edition (PLE) version 7 for windows.

The ability of the derived SIR model to reproduce the general disease incidence pattern of the Nyimba bubonic plague outbreak for the period of initial growth of the outbreak up to its peak was assessed. This was done to optimize and validate the model through model simulation against the actual outbreak data. The model simulation was done using the systems dynamics modeling software Vensim—personal learning edition (PLE) version 7 for windows [48–50]. Fig 2 shows the flow diagram of the human population system for the derived SIR model for bubonic plague infection as was generated for the model simulation.

In the diagram (Fig 2), the initial population is the total number of the humans in the population (*N*_*h*_) and at the start the number of susceptible people is given as initial population minus one (the index case). This initial population is equal to the total population for the model system (total number of individuals in the compartments) at any given time (t) during the outbreak: Initial population = total population = susceptible + infectious + removed. The infection term in the diagram describes the flow of individuals from the susceptible compartment to the infectious compartment and it is given as the quantity $\frac{\beta_{c}S_{h}(t)I_{h}(t)}{N_{h}}$ in Eq 2. On the other hand, the recovery term in the diagram describes the flow of individuals from the infectious compartment to the removed compartment and is given as *αI*_*h*_*(t)* in Eq 2. For the model simulation, the general initial conditions *N*_*h*_ = 350, *S*_*h*_*(0)* = *N*_*h*_− 1, *I*_*h*_*(0)* = 1, *R*_*h*_*(0)* = 0 were used. Fixed parameter values used for the model were as given in Table 1. However, initial random parameter sensitivity analysis for the model showed that the model output was more sensitive to changes in the value of *β*_*c*_. Note that *β*_*c*_ is comprised of the parameters *β*_*f*_, *β*_*h*_ and *μ* of which *β*_*f*_, the transmission rate of bubonic plague from infectious human ectoparasites vectors to susceptible humans per unit time, was relatively the more uncertain of these sub-parameters in literature. Therefore, a narrow range of about 0.05–0.123 was considered for *β*_*f*_ as compared to the fixed value of 0.05 used for this parameter in a similar mathematical model created for bubonic plague infection by [6]. The reason for accommodating this short range for the value of *β*_*f*_ was because currently there are limited studies that have investigated the potency of these human ectoparasites (such as the human fleas and body lice) as vectors for bubonic plague infection [6,8,32]. In fact, as was clarified in their work, the value of 0.05 used for this transmission rate in the model by [6] was specific for body lice only but in their study it was taken to represent on average the rate at which all the human ectoparasites (both human fleas and body lice) transmit the infection to susceptible humans. Therefore, since this combined average transmission rate of infection from the infectious fleas and body lice to humans may not be precisely known, the range given above was used for *β*_*f*_ in this study. Consequently, for the model simulation the fixed values for the other parameters *β*_*h*_ and *μ* were maintained as given in Table 1 while different values for *β*_*f*_ were used from the possible range of values given for this parameter. Sixteen model simulations were therefore run by appropriately altering the value of *β*_*f*_ which gave different values of *β*_*c*_ per run until a value of *β*_*c*_ that produced the best model fit to the targeted Nyimba bubonic plague outbreak data was found. This was statistically confirmed using the chi-square goodness of fit test at a significance level of 0.05. The value of *β*_*c*_ which produced better model fit to the outbreak data was therefore used in the estimation of the basic reproduction number (R_0_) for bubonic plague. The model was simulated over a 45-day time period which was approximately the duration of the Nyimba bubonic plague outbreak [11]. In the model, the first day for the simulation was set as the 23^rd^ of March 2015 even though the date of onset of symptoms for the first recorded case during the actual outbreak was the 26^th^ of March 2015. This was done so as to accommodate the brief preclinical stage of the infection for the first case before this patient became symptomatic and recorded on the 26^th^ of March 2016.

### Estimating R_0_ for bubonic plague from the derived SIR model

With the initial conditions given as; *S*_*h*_*(0)*, *I*_*h*_*(0)*, *> 0*, and *R*_*h*_*(0) = 0*, the basic reproduction number (R_0_) for bubonic plague was estimated directly from the derived SIR model system of Eq 2 as ${\text{R}}_{0}=\frac{\beta_{c}}{\alpha }$ [37,44,51]. This solution for R_0_ was derived as follows:

If the infection is spreading in the population such as during an outbreak, then we expect the number of new human infections to be increasing in the population. This means that:

$$\frac{dI_{h}(t)}{dt}>0$$

Removing the *(t)* notation for convenience, this statement means that from Eq 2(b) above we have:

$$\frac{\beta_{c}S_{h}I_{h}}{N_{h}}-\alpha I_{h}>0$$

which can be re-written as

$$\frac{\beta_{c}S_{h}I_{h}}{N_{h}}>\alpha I_{h}$$

and this yields

$$\frac{\frac{1}{N_{h}}\beta_{c}S_{h}\;}{\alpha }>1$$

which we will denote as **inequality 1.**

As per definition of R_0_, inequality 1 is analyzed at the starting point of the outbreak where the entire population can still be considered to be completely susceptible except for the one infectious individual at *I*_*h*_*(0)* introduced in the population which is the index case. At this point, *N*_*h*_ ≈ *S*_*h*_*(0)*. The appropriate notation for inequality 1 at this point can therefore be given as:

$$inequality\;1\frac{\frac{1}{N_{h}}\beta_{c}S_{h}(0)\;}{\alpha }>1$$

With the conditions given at this point as *N*_*h*_ ≈ *S*_*h*_*(0)* when the index case is introduced in the population, it means that inequality 1 simplifies as:

$$\frac{\frac{1}{N_{h}\;}\beta_{c}S_{h}(0)}{\alpha }>1\Rightarrow \frac{\beta_{c}}{\alpha }>1$$

From this we have that ${\text{R}}_{0}=\frac{\beta_{c}}{\alpha }$ and it is expected to be greater than one at the disease endemic equilibrium point, EEP, where the infection persists/spreads in the population [14].

### Ethical considerations

No ethical issues were encountered as no human or animal subjects were used in this study and cases were anonymous.

## Results

### The estimated value of R_0_ for bubonic plague using the epidemic doubling time (primary method results)

**Fig 3:**
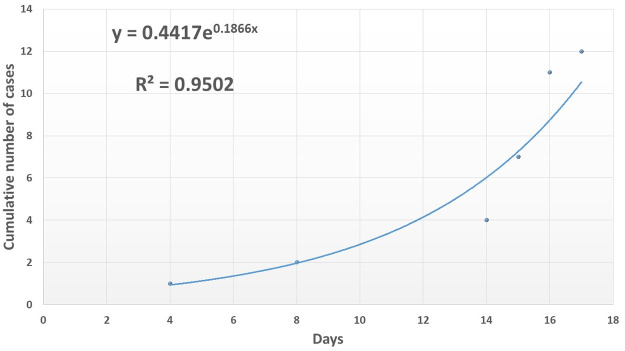
Cumulative number of cases plotted against time with fitted exponential curve for segment of initial rise of cases during the 2015 Nyimba district bubonic plague outbreak.

For the primary method, Fig 3 shows the graph of the cumulative number of cases plotted against time with fitted exponential curve for the period of initial growth of the Nyimba bubonic plague outbreak up to its peak. An R^2^ value of 0.9502 was accepted as significant for the fitted exponential curve [20]. In Nyimba, the first case was recorded on the 26^th^ of March, 2015 which corresponded to day 4 of the outbreak in the graph while the 8^th^ of April, when the outbreak was at its peak, corresponded to day 17.

**Fig 4:**
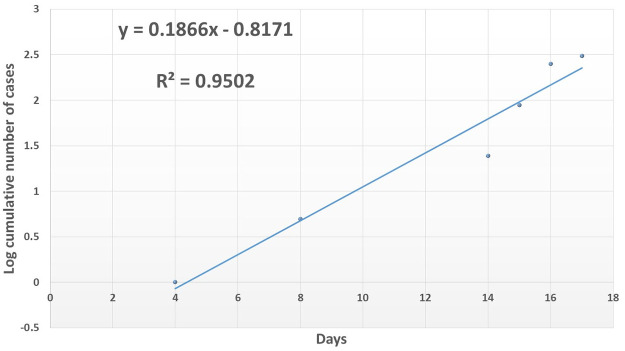
Log cumulative number of cases plotted against time and the fitted linear regression model for segment of initial rise of cases during the 2015 Nyimba district bubonic plague outbreak (R^2^ value of 0.9502 for the fitted linear model equally accepted as significant).

The fitted exponential model predicted an average initial epidemic growth rate of about 0.1866 cases/day with the 95% confidence interval estimated to be between 0.1273 and 0.2459 cases/day from the linear regression analysis of the log-transformation of Fig 3 as given in Fig 4 and Table 2 (epidemic growth rate as gradient of the slope).

**Table 2:** The linear regression output for the fitted linear regression model to the log cumulative number of cases plotted against time for segment of initial rise of cases during the 2015 Nyimba district bubonic plague outbreak.

	*Coefficients*	*Standard Error*	*t-Statistic*	*P-value*	*95% Confidence interval*	
					*Lower (95%)*	*Upper (95%)*
**Intercept**	-0.81712030	0.28203999	-2.89717890	0.04424295	-1.6001888	-0.0340518
***X* Variable**	0.18663345	0.02136095	8.73713443	0.00094552	0.12732596	0.24594094

Table 3 below shows the list of parameter estimates used to calculate R_0_ using Eq 1 and Table 4 shows the estimated value of R_0_. The range in the possible values of R_0_ is due to the estimated duration of infectiousness for bubonic plague which was used.

**Table 3:** Final list of parameters estimates used to calculate the basic reproduction number for bubonic plague using Eq 1.

Parameter	Symbol	Value	95% Confidence interval		Unit
			Lower limit	Upper limit	
*ln* 2	*ln* 2	0.693147181	N/A	N/A	N/A
Average epidemic growth rate	*α*	0.186633452	0.127325959	0.245940944	cases/day
Corresponding average epidemic doubling time	*Td*	3.713949314	2.81834805	5.443879499	days
Infectious period (lower limit)	*D*	3 [6–8,30,33,35,43]	N/A	N/A	days
Average infectious period	*D*	4 [6–8,30,33,35,43]	N/A	N/A	days
Infectious period (upper limit)	*D*	5 [6–8,30,33,35,43]	N/A	N/A	days

**Table 4:** The estimated range of the basic reproduction number for plague for the 2015 Nyimba bubonic plague outbreak.

Infectious period	The basic reproduction number (R_0_)	95% Confidence interval	
		Lower limit	Upper limit
Infectious period = 3 days	1.5599	1.382	1.7378
Infectious period = 4 days	1.7465	1.5093	1.9838
Infectious period = 5 days	1.9332	1.6366	2.2297

The basic reproduction number for bubonic plague in this study was therefore estimated to range between 1.5599–1.9332 with an average estimate of 1.7465 as shown in Table 4.

### The estimated value of R_0_ for bubonic plague from the derived SIR deterministic compartmental mathematical model (secondary method results)

**Fig 5:**
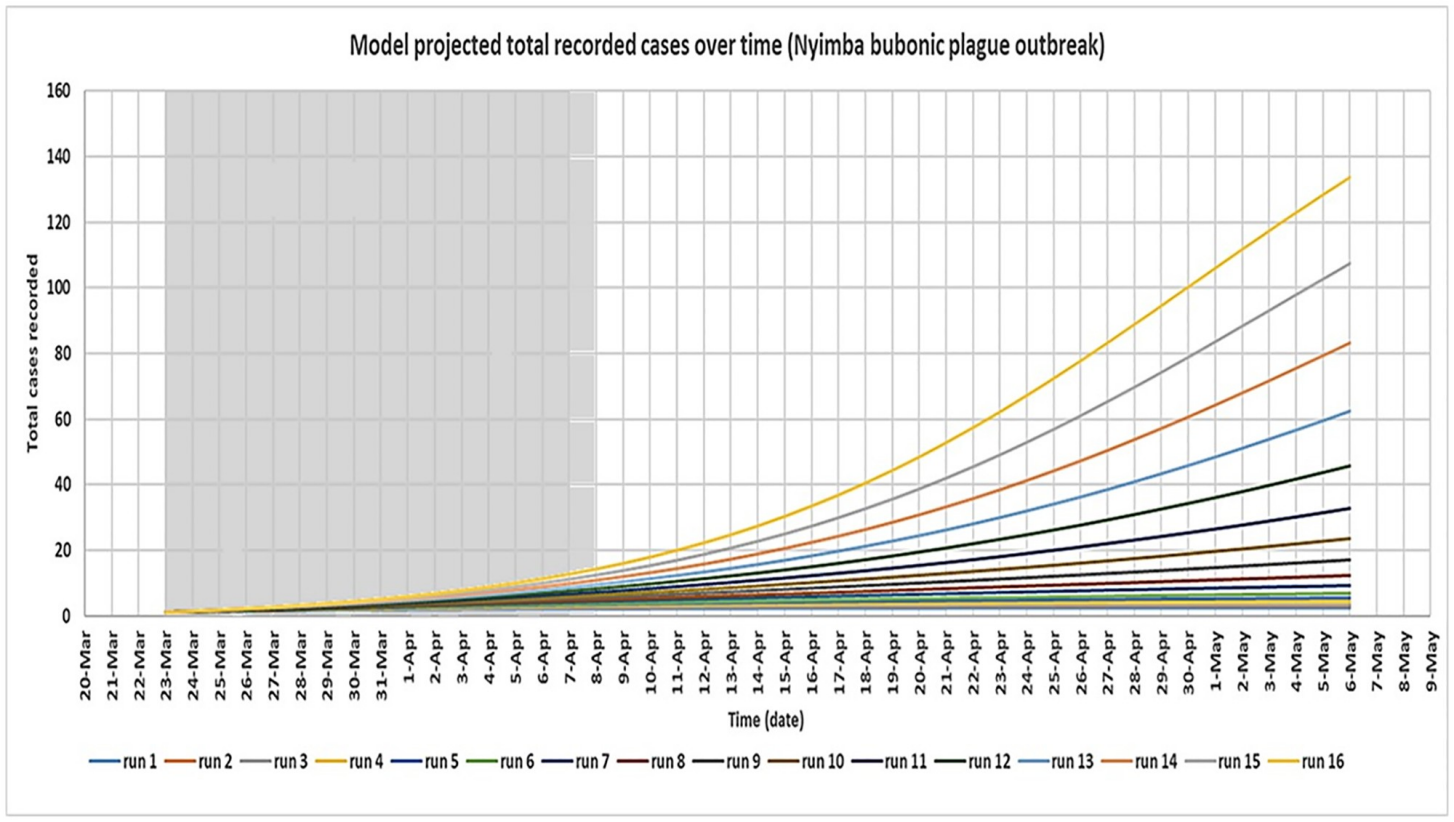
Derived SIR model simulations showing the model predicted total (cumulative) cases recorded over time for the Nyimba bubonic plague outbreak assuming no intervention was provided (model simulation over a 45-day period.

For the secondary approach, Fig 5 shows the model predicted total (cumulative) recorded cases over time for the Nyimba bubonic plague outbreak for the different runs (simulations) of the model assuming no intervention. Each separate line (simulation) in the graph represents the model output for each corresponding value of *β*_*c*_ used per simulation after altering the values of *β*_*f*_. The shaded area represents the period of initial growth of the outbreak up to its peak while the un-shaded area shows the model predicted growth of the outbreak without interventions over the projected 45-day time period.

**Fig 6:**
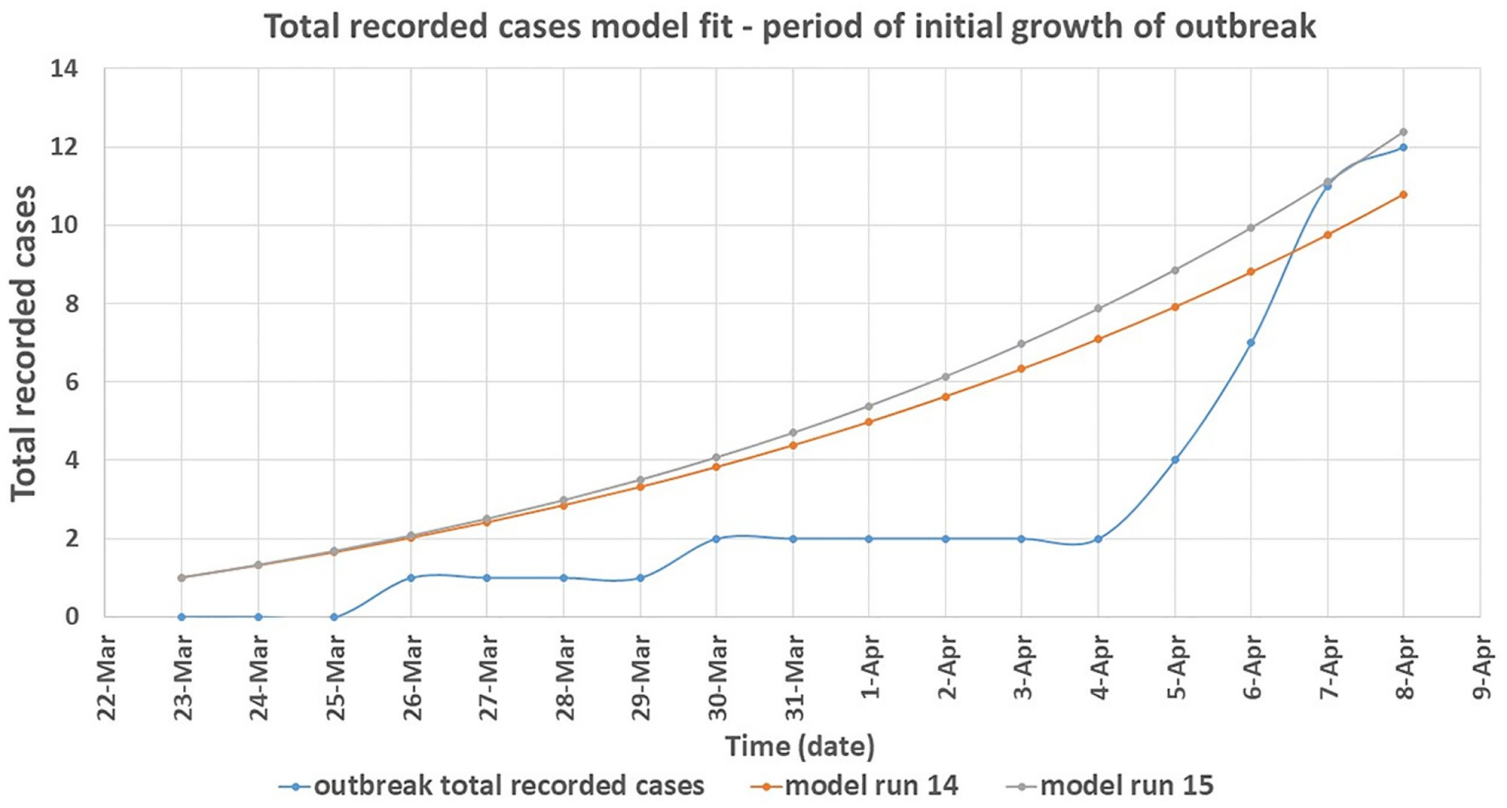
Derived SIR model simulation showing the model fit for runs 14 and 15 to the total (cumulative) number of recorded cases over time during the period of initial rise of cases for Nyimba bubonic plague outbreak. Goodness of fit: run 14 (P-value 0.202), run 15 (P-value 0.064).

**Fig 7:**
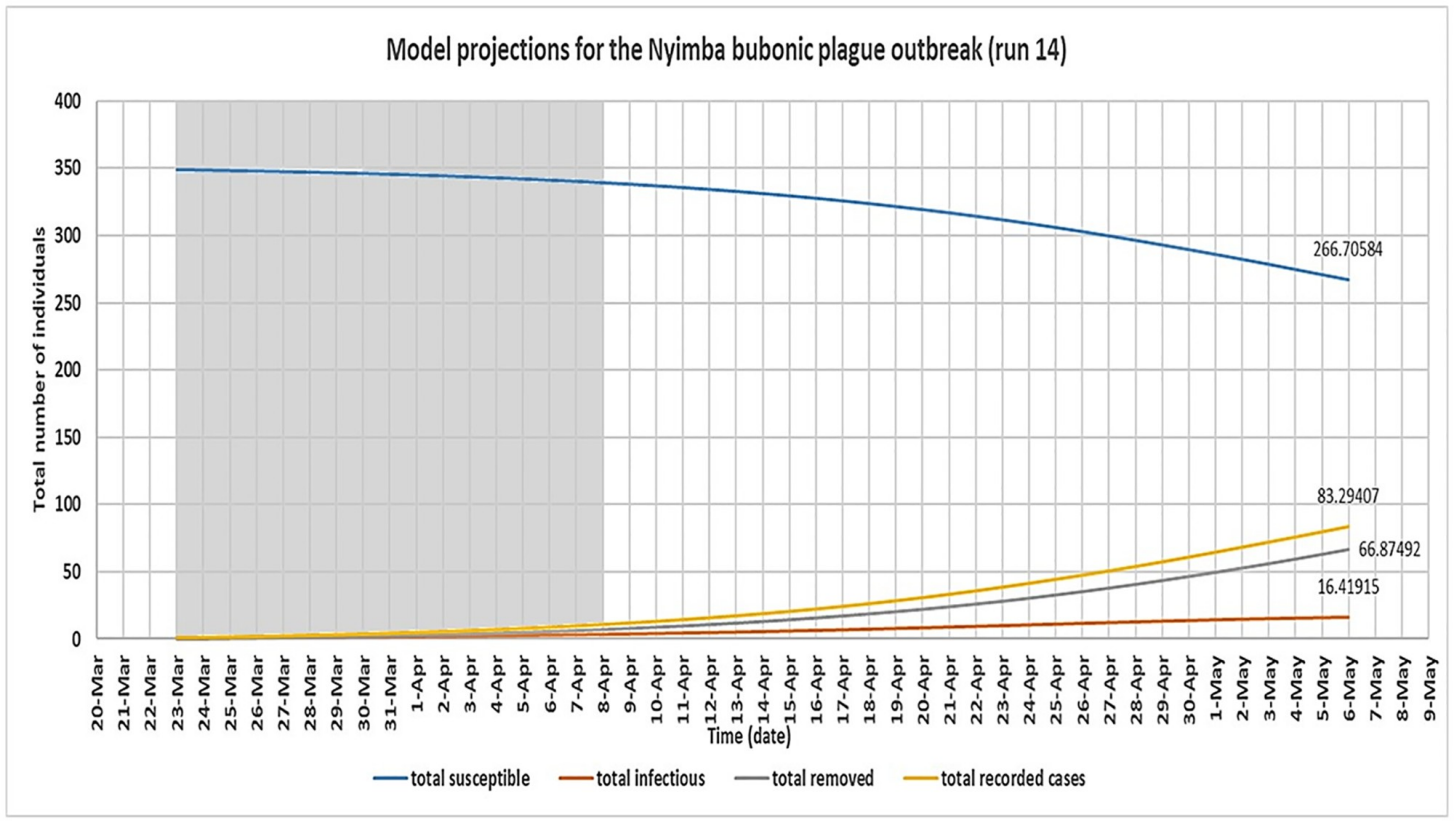
Derived SIR model simulation showing the model predicted most likely progression of the Nyimba bubonic plague outbreak for the different infection states of individuals for run 14 of the model simulation assuming no intervention was provided. (model simulation over a 45-day period).

**Fig 8:**
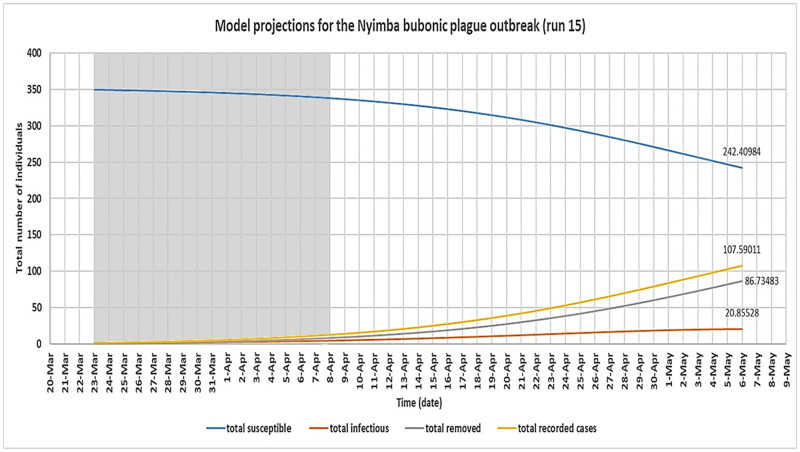
Derived SIR model simulation showing the model predicted most likely progression of the Nyimba bubonic plague outbreak for the different infection states of individuals for run 15 of the model simulation assuming no intervention was provided. (model simulation over a 45-day period.

The optimal configuration of the model was determined by analysing the outbreak data for the period of initial growth of the outbreak up to its peak (shaded area). This is because it was expected in this study that the disease transmission dynamics occurred naturally during this period of the outbreak; an assumption implicit in the model design. Therefore, the model predicted cumulative number of cases over time for each simulation for this period was compared to the actual numbers recorded during the Nyimba outbreak for the same period. This was done for all dates on which new cases of bubonic plague were recorded within this period which are the 26^th^ of March, 30^th^ March, 5^th^ April, 6^th^ April, 7^th^ April and 8^th^ April 2015 [11]. These dates correspond to day 4, day 8, day 14, day 15, day 16 and day 17 respectively of the model simulation. As seen in Table 5, the corresponding actual cumulative number of cases that were recorded during the Nyimba outbreak on these days were: day 4 = 1, day 8 = 2, day 14 = 4, day 15 = 7, day 16 = 11 and day 17 = 12 (red colour). Considering the whole number values of the model predicted total cases over time, it was found that runs 14 and 15 of the model simulation (shaded in Table 5) together had the closest fit to the outbreak data for the selected dates; P-value 0.202 and 0.064 respectively (Fig 6). Therefore, the values of *β*_*f*_ on these runs of the model simulation were taken to be the most optimal estimates for this parameter based on the available outbreak data for the Nyimba bubonic plague outbreak. The corresponding values of *β*_*c*_ for these estimated values of *β*_*f*_ therefore gave the estimates of R_0_ for bubonic plague as R_0_ = 1.4 for run 14 and R_0_ = 1.5 for run 15 based on the formula ${\text{R}}_{0}=\frac{\beta_{c}}{\alpha }$. Figs 7 and 8 give the most likely progression of the Nyimba outbreak without intervention for the different infection states of individuals for the entire projected 45 day period for runs 14 and 15 of the model simulation respectively. It can be noted from these graphs that according to the model predictions, the infections in the human population during the Nyimba outbreak could have continued to rise and peak with more than 80 total recorded cases about 45 days later had it not been for the prompt action and interventions initiated by the Nyimba district health team, Zambia Ministry of Health, and other stakeholders that took action [11].

**Table 5:** The model predicted cumulative cases on day 4, 8, 14, 15, 16, and 17 for the 2015 Nyimba bubonic plague outbreak based on the different values of *β*_*f*_ and *β*_*c*_ and the corresponding estimates of R_0_ (Data generated in Vensim systems dynamics modeling software—Personal learning edition. R_0_ values calculated in Excel).

			Day 4	Day 8	Day 14	Day 15	Day 16	Day 17	
*β*_*f*_	*Β*_*c*_	Outbreak total cases	1	2	4	7	11	12	R_0_
		Model total cases							
0.050	0.138	Model predicted case incidence for Run 1	1.3772	1.7458	2.1016	2.1445	2.1837	2.2195	0.6188
0.055	0.151	Model predicted case incidence for Run 2	1.4195	1.8505	2.2965	2.3532	2.4058	2.4545	0.6789
0.060	0.164	Model predicted case incidence for Run 3	1.4630	1.9624	2.5164	2.5906	2.6603	2.7259	0.7391
0.065	0.178	Model predicted case incidence for Run 4	1.5075	2.0822	2.7646	2.8607	2.9524	3.0398	0.7993
0.069	0.191	Model predicted case incidence for Run 5	1.5531	2.2101	3.0450	3.1685	3.2879	3.4033	0.8594
0.074	0.204	Model predicted case incidence for Run 6	1.5998	2.3469	3.3618	3.5193	3.6736	3.8247	0.9196
0.079	0.218	Model predicted case incidence for Run 7	1.6476	2.4929	3.7197	3.9192	4.1172	4.3137	0.9798
0.084	0.231	Model predicted case incidence for Run 8	1.6965	2.6488	4.1242	4.3751	4.6275	4.8811	1.0400
0.089	0.244	Model predicted case incidence for Run 9	1.7466	2.8152	4.5811	4.8950	5.2147	5.5400	1.1001
0.094	0.258	Model predicted case incidence for Run 10	1.7979	2.9926	5.0973	5.4877	5.8901	6.3048	1.1603
0.099	0.271	Model predicted case incidence for Run 11	1.8503	3.1817	5.6800	6.1631	6.6670	7.1924	1.2205
0.103	0.285	Model predicted case incidence for Run 12	1.9040	3.3832	6.3376	6.9322	7.5599	8.2219	1.2807
0.108	0.298	Model predicted case incidence for Run 13	1.9588	3.5978	7.0791	7.8077	8.5854	9.4149	1.3408
0.113	0.311	Model predicted case incidence for Run 14	2.0148	3.8263	7.9148	8.8032	9.7620	10.7959	1.4010
0.118	0.325	Model predicted case incidence for Run 15	2.0721	4.0693	8.8556	9.9341	11.1106	12.3923	1.4612
0.123	0.338	Model predicted case incidence for Run 16	2.1306	4.3278	9.9137	11.2174	12.6540	14.2348	1.5214

### Discussion

In this study, the basic reproduction number (R_0_) for the most recent outbreak of bubonic plague which occurred in 2015 in Nyimba district of Eastern province in Zambia was estimated. To estimate this R_0_, a simplified robust mathematical method that estimates R_0_ directly from epidemic incidence data was used (primary method) in light of the limited epidemiological data for the study outbreak [11, 12–14,19,20,39]. R_0_ for bubonic plague was estimated to be effectively above unity ranging from 1.5599–1.9332 with an average of 1.7465. The Nyimba plague outbreak reached its peak as early as around the 7^th^– 8^th^ of April 2015 after which it was generally brought under control with the last case recorded on the 1^st^ of May 2015 [11]. However, it is the data from the initial exponential growth phase of the epidemic that the basic reproduction number was estimated i.e. when the population was still considered to be completely susceptible.

In the first instance, the R^2^ value of 0.9502 for the fitted exponential curve to the observed early epidemic incidence data in the study was taken to validate the assumption of initial exponential growth of the outbreak. This assumption is further upheld by the estimated value of R_0_ that is entirely above unity as has been reported in other studies [29]. Further, it was noted that the main victims of this outbreak were children of which about 95% were below the age of 15 years as previously stated [11]. Children of this age, especially in remote villages, usually come together to play through various activities allowing for frequent random contact and homogenous mixing amongst themselves. Therefore, after introduction of the infection into the population, most likely through an epizootic spill over event, it is highly possible that it was during such events of interaction that some infected children may have spread the infection to other susceptible children. It is suspected that the ordinary human ectoparasites (such as human fleas and body lice) may have played a major role as vectors in the transmission of the infection. This is because human ectoparasites are believed to be capable of transmitting plague [6–8,31,34,35]. A recent study in Zambia [52] reported that plague occurs more frequently in poorly maintained households with dirty surroundings and since the outbreak location in this study is a crowded village setting, it is highly likely that the victims harbored human ectoparasites. In fact, it is usually expected that hygiene standards are lower in children and young adolescents as compared to adults and therefore these children could have indeed carried various human ectoparasites that could spread the infection.

The estimated value of R_0_ for bubonic plague in this study shows that the disease is capable of epidemic spread if it occurs in susceptible populations in the country [13,14,18,21]. We found that each infectious individual may infect up to two other susceptible individuals in a worst case scenario (depending on the infectious period and other population parameters). During the Nyimba outbreak, the first two cases appeared on the 26^th^ and the 30^th^ of March 2015 [11]. After this, a period of about 5 to 9 days passed before a large cluster of cases (9 new cases) suddenly appeared again around the 5^th^–7^th^ of April 2015 (peak period) [11]. It is therefore possible that those initial captured cases, and possibly other uncaptured cases, may have given rise to some of those new cases after the gap period if one considers the assumed infectious period for bubonic plague, a possible brief pre-clinical stage, and the estimated R_0_ for the infection in this study [7,8,53]. This may not be the exact evolution of the events during the Nyimba outbreak but it is very plausible based on the estimations from this study.

A simple SIR deterministic compartmental mathematical model for bubonic plague infection was also developed in this study. As seen in Table 5 (runs 14 and 15), the model was generally able to fit the outbreak data for the Nyimba outbreak in terms of the cummulative number of cases recorded over time for the period of initial growth of the outbreak. The success of this model, despite its limitations, in fitting and reproducing the general spread pattern for the Nyimba outbreak provided the opportunity to use it as a secondary approach for estimating the basic reproduction number for plague in order to give credence to the results of the first and primary method for this study. The basic reproduction number for bubonic plague was therefore estimated from the model to be about 1.4 to 1.5. This value of R_0_ was slightly lower but still fell well within the 95% confidence interval of the estimated range for R_0_ using the primary method. This outcome was taken to validate the results of this study to some extent since there was a general consistency in the estimates of R_0_ for the same bubonic plague outbreak despite using two very different methods of estimation. This observed “agreement” in the two results (although not a perfect agreement) is despite the uncertainties associated with estimating R_0_ using various parameters of a compartmental model. For example, in this model used, all the parameters (*β*_*c*_ and α) on which R_0_ depends generally had to be estimated from literature which can introduce unavoidable uncertainty in the R_0_ estimate because these parameter values also greatly vary in literature. For this study, this was of great significance because changes in the composite parameter *β*_*c*_ (which comprised of various parameters estimated from literature) had the greatest effect on changes in both the model predicted number of cases over time and the estimated value of R_0_ for bubonic plague. Other parameter values were kept constant for simplification of the model.

The estimated value of R_0_ for bubonic plague in this study was also found to be essentially within the same range as that estimated for previous rapid spreading bubonic plague outbreaks of the 14^th^ century Black Death plague in Europe for which R_0_ was estimated to range between 1.5 and 1.9 [6]. As is generally known, medieval bubonic plague outbreaks from the 14^th^ to 18^th^ century, such as the well-known Black Death, were documented to have been more devastating, with higher attack rates than modern day third pandemic outbreaks [6,31,53]. The similar R_0_, but different magnitude of outbreaks between our study outbreak and previous pandemics is most likely due to similar transmission patterns but present day treatment and containment measures reducing the susceptible population fast [1,6,31,54]. This could be the reason for the observed difference in the magnitude of outbreaks seen between these pandemics despite having a similar estimated R_0_ for the infection. The result of this study therefore puts forward some scientific evidence that bubonic plague outbreaks today are still inherently capable of widespread transmission with devastating effects if left unchecked. In the case of Zambia, this raises particular concern considering that starting from about 1997 there has been a sudden occurrence of large plague epidemics than ever recorded before in the country [1]. The reason for these seemingly “more intense” plague outbreaks is not well known but may point to delayed intervention. Recent studies in Zambia however found that recorded heavy rainfalls due to climate change in the recent decades and certain social cultural factors including lifestyle practices may have contributed to the occurrence and rapid spread of the infection during the outbreaks. Practices such as wild rodent bush hunting and bush cattle rearing, movements of people from infected to uninfected areas, polygamy with husbands spreading infection between villages, beliefs in witchcraft as cause of disease resulting in delayed diagnosis, overcrowding and sleeping on floors in poorly maintained households were all among the chief factors identified in these studies [1,9–11]. By estimating R_0_, this current study was able to attach a quantitative aspect to describing and understanding the possible transmission dynamics of bubonic plague in a local setup in Zambia. With this new added knowledge of R_0_, we can now use this estimate to predict the size and progression of future outbreaks and quantitatively assess the effects of various control measures required to prevent the spread of infection through further mathematical modelling techniques [13,14,24–28,37,55]. For example, one simple and classical demonstration of the invaluable use of R_0_ is that it is used to determine the minimum mass treatment or vaccination coverage rate required to prevent spread of the infectious disease in a population [13,37]. Therefore, based on the average value of R_0_ estimated in this study, a minimum vaccination (or prophylactic treatment) coverage rate of about 43% would be enough to prevent the spread of bubonic plague infection in an identified risk population [13,18,21,37]. Further, it is clear from the expression of R_0_ in the secondary method, that the disease will die out if *β*_*c*_ < α, i.e., if the recovery rate is greater than the composite human to human transmission rate, as expected. Finally, similar to the conclusions by [6], the results of this study suggest that ectoparasitic transmission of bubonic plague may be more likely than previously thought. By using two methods to estimate R_0_, the study shows that the plague outbreak in Nyimba followed an exponential growth rate suggestive of human to human transmission. This implies that a human to human plague transmission pathway via ectoparasites has to be considered under the present conditions. With socio-cultural and ecological changes in plague endemic developing countries, this transmission pathway could become more prominent and adequate control methods should be employed.

It is noted however that the result of this study may not be readily representative for all recent bubonic plague outbreaks seen in Zambia since the value of R_0_ may vary from one area to another depending on population dynamics and other factors. Additionally, it is also acknowledged that the methods used here for estimating R_0_ are still subject to certain weaknesses most important of which is the completeness and quality of the reported epidemic incidence data used for the analysis. For example, 29 out of all the 111 patients suspected to have bubonic plague during the Nyimba outbreak were not available for physical examination [11]. This could have caused the value of R_0_ to be underestimated in this study as it is generally possible that some cases during the outbreak may have been misdiagnosed, never reported or even died before they went to the hospital. This could have particularly affected the result from the model as it is possible that the model used in this study may have been fit to data that was incomplete. Another limitation of the study is the small sample size available for the analysis despite use of a robust method of estimating R_0_ as well as the limited background data about the affected population. More similar studies with larger sample sizes will need to be conducted. In spite of this, it is the researcher’s recommendation that the estimate of R_0_ for bubonic plague obtained in this study can still be used as a primary reference value in the analysis of future outbreaks of the disease in similar populations in the country especially in emergency situations. Alternatively, this study has demonstrated the practical use of a quick and robust method for estimating R_0_ directly from outbreak incidence data for an infectious disease such as bubonic plague. Therefore, this method can still be employed in future to obtain an updated estimate of R_0_ during the early stages of an outbreak of bubonic plague or other infectious diseases using the outbreak incidence data.

### Conclusions

The basic reproduction number for the 2015 Nyimba district bubonic plague outbreak was estimated to range between 1.5599 and 1.9332 with an average of 1.7465. This value of R_0_ which is above unity is a quantitative indicator that indeed bubonic plague is capable of epidemic spread in Zambia as was seen in Nyimba. The value of R_0_ estimated here also suggests that there is a present potential threat of the occurrence of large bubonic plague outbreaks in Zambia if conditions become favorable.
